# Livelihood vulnerability of rural households to climate variability and change: An agroecological system-based approach in northwestern Ethiopia

**DOI:** 10.1016/j.heliyon.2024.e40570

**Published:** 2024-11-20

**Authors:** Tatek Belay, Tadele Melese Lebeza

**Affiliations:** a Department of Geography and Environmental Studies, College of Social Science and Humanities, Debre Tabor University, P.O. Box 272, Debre Tabor, Ethiopia; b Department of Natural Resource Management, College of Agriculture and Environmental Science, Bahir Dar University, P.O. Box 5501, Bahir Dar, Ethiopia

**Keywords:** Adaptative capacity, Climate change, Exposure, Livelihood, Sensitivity, Vulnerability index

## Abstract

Ethiopia is widely acknowledged to be extremely vulnerable to climatic variability and change. Its agricultural sector, which is particularly susceptible to risks associated with rainfall variability, represents a major source of vulnerability. Household livelihood vulnerability varies across different agroecological zones (AEZs). This research aimed to investigate farmers' susceptibility to climate variability and change in northwestern Ethiopia. A total of 405 households from three AEZs (lowland, highland, and midland) were chosen through simple random sampling. Focus group discussions and key informant interviews were also conducted to complement and validate the numerical data. To assess the household vulnerability levels, the social, economic, and environmental indicators were aggregated, and the LVI and LVI-IPCC index were employed. The study's findings indicated significant variation in livelihood vulnerability indices, profiles, and indicators across agroecological zones (AEZs). The midland AEZ exhibited low exposure, higher adaptive capacity, and low vulnerability, while the lowland AEZ showed higher vulnerability, weaker adaptive capacity, and greater exposure compared to the midland and highland AEZs. The LVI-IPCC analysis corroborated these findings, with values of 0.128 for the midland AEZ, 0.168 for the highland, and 0.180 for the lowland. The variation is due to differences in agroecological and socioeconomic factors among households. The results indicate the necessity of implementing adaptation strategies specifically tailored to the agroecological systems in the study area. Such an approach is essential for effectively reducing vulnerability among households engaged in mixed crop-livestock agriculture. Adaptation measures developed from these assessments are both applicable and relevant to local conditions, as they are grounded in the community’s long-term realities.

## 1 Introduction

Global warming and its effects, such as climate extremes and associated risks, have a negative influence on livelihoods and human well-being in many regions of the world [1]. Rural communities in developing nations face serious challenges to their livelihoods from climate variability and change (CVC). The impact is notably severe in Africa due to a rising dependence on natural resources and rain-fed agriculture in response to a changing climate [2].

The vulnerability to CVC in African countries often coincides with poverty, resulting in significant challenges to people's livelihoods [3,4]. The unpredictable shifts and geographical disparities in climate variables create uncertainty and strain on agricultural activities, which in turn affect livelihoods. Small-scale farmers, relying significantly on rain-fed and climate-vulnerable agriculture, are among the most affected [5]. These farmers can only thrive with their ordinary farming practices and livelihood strategies under favorable temperature and rainfall conditions.

The results of smallholder farmers' livelihood activities are greatly impacted by their incapacity to adapt to CVC [6,7]. It is possible to improve livelihoods, household income, and well-being, decrease vulnerability, and encourage the sustainable use of natural resources by recognizing the effects of climate change and changing existing habits. While smallholder farmers’ livelihoods have long faced numerous challenges, climate change not only brings new challenges but also exacerbates existing bottlenecks, making it increasingly difficult to meet livelihood needs. Regulating climate change brings an opportunity to address these problems for the betterment of individual farmers and the community.

It is commonly acknowledged that one practical approach to reducing climate change and achieving sustainable development is climate adaptation [8]. Given the substantial effects of climate change on smallholder farmers' lives, it is critical to promote adaptation and guarantee that adaptation plans are successful in maintaining their standard of living.

Ethiopia is particularly susceptible to changes in the climate [9]. One of the main causes of this vulnerability is the population's heavy reliance on rain-fed agriculture [10], together with high rates of natural resource degradation, rapid population increase, pervasive poverty, and a lack of institutional and socioeconomic capacity to handle risks and hazards [3,11]. The vulnerability to climate extremes and hazards results in droughts that impact both crop and livestock production when there is irregular, unpredictable, and insufficient rainfall [12].

Inadequate and erratic rainfall patterns in the majority of Ethiopia have an impact on local livelihoods and result in low agricultural yields [13]. Smallholder farmers experience both short-term and long-term food instability and hunger as a result of their reliance on rain-fed agriculture [14,15]. To effectively mitigate and manage these risks, long-term and sustainable disaster risk reduction strategies should be community-based, taking into account the specific vulnerabilities of smallholder farmers to climatic extremes [15]. In Ethiopia, farmers utilize various adaptation strategies to improve their resilience, including adjusting cropping practices, managing resources, and diversifying into non-farm activities [14,16].

A solid understanding of risk assessment and practical climate change solutions are critical to promoting sustainable development in the farming sector in the Amhara region. The effects of Ethiopia's susceptibility to different climatic pressures have not yet been well studied, which makes it difficult for policymakers to create viable plans for sustainable livelihoods [12].

Participating in alternative livelihood activities might improve adaptive capability by providing households with an alternative source of income in the event of climatic extremes [17]. Various communities and areas may experience varying impacts from climate stress based on their sensitivity and ability to respond [18]. Thus, even within the same area, communities may have varying drought sensitivities despite facing similar exposure to climate stress. The main causes of increased livelihood vulnerability are high sensitivity and weak adaptation capability [19].

Improving agricultural systems' ability to adapt to climate change is crucial, as adverse weather conditions are anticipated to increase in frequency [6]. Numerous empirical studies conducted worldwide have shown that building resilience to climate change in agriculture ensures the sustainability of agricultural-based livelihoods and reduces vulnerability to climate-related stress [17,20]. To decrease the adverse effects of climatic extremes, several kinds of adaptation strategies have been implemented [21,22]. Moreover, context-specificity is essential for climate change adaptation and resilience [6].

Numerous factors can impact resilience and climate adaptation, including household income, diversity of livelihoods, geography, environmental perspectives, and ecological, human, and socioeconomic aspects [[23], [24], [25]]. In Ethiopia, smallholder farmers who depend on agriculture as their primary source of income are especially vulnerable to the effects of CVC [26,27]. In some parts of Ethiopia, farmers have diversified their livelihoods as an adaptation strategy to cope with climate change and its associated stressors such as erratic rainfall, higher temperatures, and prolonged droughts [28,29].

A comprehensive study of sustainable livelihood development has been carried out by researchers, who have integrated vulnerability factors and livelihood approaches. The elements that contribute to overall livelihood vulnerability have been the subject of numerous research conducted in Africa and other locations, with differing findings [20,30]. Studies conducted in Africa indicate that livelihood vulnerability is largely caused by a lack of adaptation capacity and increased exposure to climate-related risks, like drought [16,29]. Thus, the sustainability of the livelihoods of small-scale farmers is compromised. Notably, Hahn et al. [31] assessed community livelihood vulnerability using the Sustainable Livelihoods approach and the IPCC-LVI (Livelihood Vulnerability Index) to provide empirical knowledge. The LVI-IPCC technique has been used in Ethiopia and, it serves as the basis for this research as highlighted by earlier studies [[32], [33], [34], [35]].

Earlier studies in Ethiopia were conducted at a macro level [3,15,36], making them less applicable for designing adaptation-mitigation projects or local-level planning. Examining and assessing the effects of climate change requires a context-specific approach that takes into account local realities and socioeconomic conditions. This method is essential to develop strategies and policies that can reduce the negative impacts on livelihoods. This allows for the development of appropriate coping and adaptation techniques to lessen smallholder farmers' susceptibility to climate change impacts.

In Ethiopia, several studies have looked at the factors that lead to vulnerability as well as the impact of climate variability and change to improve a household’s capacity and to support a livelihood. Some studies have used the equal-weighting approach [[37], [38], [39]]. However, it is important to note that not all vulnerability variables contribute equally to vulnerability [32,34]. Furthermore, studies conducted in Ethiopia revealed conflicting findings about the factors that contribute to vulnerability, indicating that location affects vulnerability. For example, studies conducted by Asfaw et al. [16], Maru et al. [40], and Tessema & Simane [39] have shown that communities living in lowlands are more susceptible to hazards associated with climate change than communities in other AEZs. However, Asmamaw et al. [41] and Simane et al. [15] found that highland areas are more vulnerable to livelihood strategies than lowland and midland regions. Additionally, midland regions are more vulnerable than highland regions [19]. This study aims to investigate vulnerability in a context-specific way because of the discrepancies in the results of earlier research and the requirement for an accurate evaluation of vulnerability elements.

Erratic rainfall patterns and frequent droughts are common climatic challenges in the South Gondar administrative zone. However, no prior study has been done on how vulnerable households' livelihoods are and how this susceptibility differs among societies residing in the South Gonder Zone's three agroecological zones. To address these gaps, this study aims to investigate the livelihood vulnerability of rural households to climate variability/change across three agroecological zones in Northwestern Ethiopia. The specific focus was on households in the districts of *Ebinat*, *Lay Gayint*, and *Sede Muja* were the specific focus of the study. The substantial climate vulnerability faced by households, combined with a lack of prior research on this particular region, highlights the importance of this study.

This study is highly valuable for both governmental and non-governmental organizations dedicated to improving the livelihoods and well-being of households across three AEZs. The study’s findings can identify high-risk areas, rank them, and highlight factors contributing to household vulnerability. This information will aid practitioners and policymakers in formulating effective, long-term adaptation strategies. Moreover, it will empower farmers and local government officials to develop and implement practical livelihood strategies and adaptive actions to mitigate the impacts of climate-related risks on households within the study area and similar regions. NGOs and development organizations can also use the results to target areas requiring support due to climate-related hazards.

## 2 Materials and methods

### 2.1 Description of the study area

**Fig. 1:**
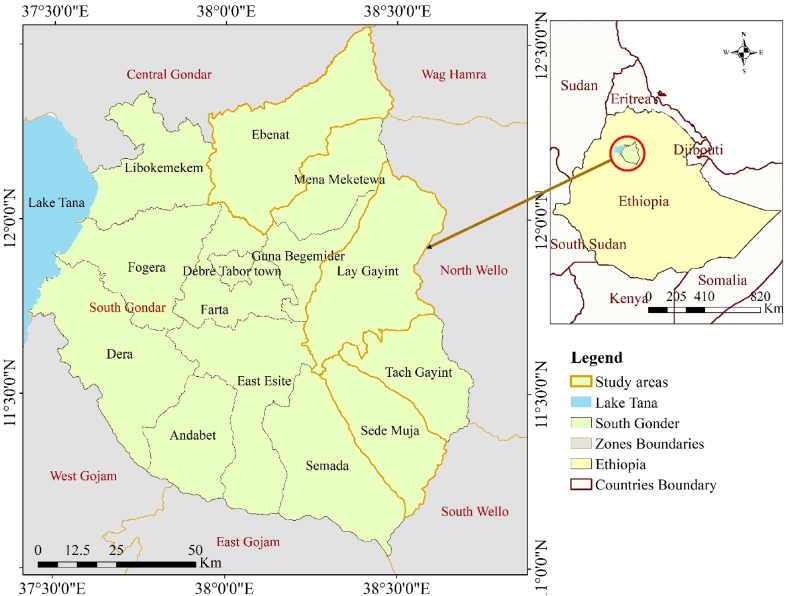
Location of the study area.

The South Gonder Administrative Zone is situated in northwestern Ethiopia. Covering an area of about 14095.44 km^2,^ it is comprised of 13 districts. It is located between 11°02'12'' and 12°33'11'' N, and 37°25'31'' and 38°43'25'' E (Fig. 1). The study was conducted in the *Lay Gayint, Ebinat*, and *Sedei Muja* districts of South Gonder Administrative Zone. These districts represent highland (2300–3200 m.a.s.l), midland (1500–2300 m.a.s.l), and lowland (500–1500 m.a.s.l) AEZs. The study area is geographically situated in the western highlands of Ethiopia, characterized by rugged topography and relatively low-lying plains. The slope gradient ranges from low to extremely steep. The sub-watersheds under study contribute to the drainage systems of the Abay and Tekeze basins.

Rain-fed agriculture is the primary source of income and livelihood. A prevalent mixed crop-livestock production system, mainly for subsistence, characterizes the region. The area experiences two distinct rainy seasons: agriculture primarily relies on the summer, locally known as the *Kiremt* season, which spans the long rainy period from June to September. Additionally, the spring season, referred to locally as *Belg* season and occurring between March and May, is also vital for agricultural activities. The area receives an average of 889 to 1257 mm of rainfall per year. The maximum temperature in the area ranges from 23.5 °C to 24.7 °C, while the minimum temperature ranges from 13.4 °C to 16.7 °C, based on data recorded between 2010 and 2020.

In the highland regions, the primary crops cultivated include highland pulses, barley (*Hordeum vulgare*), teff (*Eragrostis tef*), faba beans (*Vicia faba*), peas (*Pisum sativum*), and wheat (*Triticum aestivum*). Extensively grown in the midland areas are maize (*Zea mays*), teff (*Eragrostis tef*), sorghum (*Sorghum bicolor*), wheat (*Triticum aestivum*), chickpeas (*Cicer arietinum*), haricot beans (*Phaseolus vulgaris*), and oilseeds. Lowland regions are predominantly planted with cowpeas (*Vigna unguiculata*), sorghum (*Sorghum bicolor*), groundnuts (*Arachis hypogaea*), sunflowers (*Helianthus annuus*), teff (*Eragrostis tef*), and lentils (*Lens culinaris*). Livestock plays a crucial role in plowing, threshing, and transportation and serves as a vital source of food and household income. The study area is subjected to periodic extreme events, and the climate is variable. The study area is subjected to periodic extreme events, and the climate is variable. Food security has become a major concern due to vulnerability to climate change-related threats, along with socio-economic challenges such as limited farmland and constrained access to improved agricultural technology.

### 2.2 Research design and data collection methods

In this study, a combination of longitudinal and cross-sectional research designs were used. A multistage sampling was employed to choose the target households. The study area was stratified into three agroecological zones: highland/*dega*, midland/*weyina dega*, and lowland/*kolla*. Three districts were purposively selected: *Sedie Muja* (lowland), *Ebinat* (midland), and *Lay Gayint* (highland). The ongoing need for food assistance in the area, indicative of persistent poverty and food insecurity, served as one of the selection criteria. In the Amhara region, 48 of the 105 districts are vulnerable to drought and regularly face food shortages [42]. Thirdly, Ethiopia's lowest administrative divisions, known as kebeles, were listed and clustered according to the traits that best suited each sampled district. To exclude *kebeles* that did not fit with the predominant characteristics of the sample districts.

Six kebeles were then selected using stratified sampling, with two kebeles selected from each sampled district. *Moseb Terara* and *Ageregenet kebeles* were chosen from the highlands, *Aderseg and Abina* kebeles from the midlands, and *Densa* and *Tara* kebeles from the lowlands. Lastly, a lottery method was employed to select sample household heads from the chosen kebeles. The sampling frame consisted of a list of households residing in each kebele. The sampling frame for this study was based on a list of households from each kebele, derived from the roster of household heads in the respective kebeles. 410 households were sampled using Kotari’s formula [43] and distributed proportionally across the three agroecological zones. Thus, simple random sampling was employed to select 154, 143, and 113 households from the highlands, midlands, and lowlands, respectively.

### 2.3 Data sources and collection methods

For this study, a mix of primary and secondary data sources was used. Focus group discussions (FGDs) and pre-tested structured questionnaires were employed to gather primary data. Questionnaires were used to collect information on the socio-economic, biophysical, and institutional aspects of the study site. The main data collection tool was the household survey. A literature review was carried out to identify the principal components and sub-components of each key element to ensure alignment with the local context. Based on this, survey questions were developed to gather information on the major and sub-components of vulnerability. Each of the eleven profiles used to determine the Livelihood Vulnerability Index (LVI) was covered in the questionnaire. The details of the last three sections are provided in Table 1.

**Table 1:** The major components, sub-components, and their potential impact on vulnerability.

Factor	Major Components	Sub-component	Unit	Source
Exposure	Climate variability & change	Mean SD of average monthly maximum temperature (^o^C) (1990–2022)	^o^C	[32,35,39]
		Mean SD of average monthly minimum temperature (^o^C) (1990–2022)	^o^C	
		Mean SD of average monthly rainfall (mm) (1990–2022)	mm	
		Households who perceive increasing temperature trends	%	
		HHs who perceive a decrease in rainfall coverage during the rainy season	%	
	Natural disaster	HHs who perceive the occurrence of drought over 20 years.	%	[44,45]
		HHs who perceive delayed onset and early cessation of rainfall	%	
		HHs who reported crop disease/pest outbreaks over 20 years.	%	
		HHs who reported livestock disease outbreaks over 20 years.	%	
		HHs lack prior warning about natural disasters.	%	
Sensitivity	Agricultural system and natural resource	Crop Diversification Index	1/#crops	[16,32,35]
		Households who perceive rapid natural vegetation conversions	%	
		HHs with fertile soil	%	
		HHs who have land tenure insecurity feeling	%	
		HHs who perceive rapid land degradation	%	
		Landholding size	ha	
	Water	HHs who reported water conflicts	%	[32,46,47]
		HHs who utilize natural water sources	%	
		HHs who use a natural water source	Minute	
		Average distance to a water source by foot	%	
Adaptive capacity	Knowledge/Skill	HHs who received training to cope with climate change	%	[16,32]
		HHs not received health extension service.	%	
		HH who did not have training in farm management	%	
	Livelihood strategies	HHs practicing diversifications and coping strategies	%	[44,45]
		HHs who do not work in off-farm income activity	%	
		HHs using improved livestock bread and technology	%	
		HHs whose main sources of income are only rain-fed agriculture	%	
		HHs applying fertilizer and compost or manure	%	
		HH who believed they were experiencing food insecurity	%	
	Socio-demographic profile	Age of HH head	Years	[35,38,45]
		HH age-dependency ratio	Ratio	
		HHs with female heads	%	
		HH heads who have not attended school	%	
		Farm experience	Years	
		Family Size	Count	
	Wealth and Income	Total Tropical Livestock Unit (TLU)	TLU	[35,47,48]
		HH did not possess a home made of corrugated iron and wood	%	
		Total income from crop production	ETB	
		Total non-farm income	ETB	
	Technology	Improved seed users	%	[16,35]
		HHs with users of artificial fertilizers	%	
		Insecticide and pesticide users	%	
		HHs who don't use rain-water harvesting	%	
		HHs who don't practice irrigation	%	
		HHs who don't no access to radio	%	
	Infrastructure	Walking distance to main weather roads	Minute	[32,35,46]
		Walking distance to school	Minute	
		Walking distance to extension services	Minute	
		Walking distance to a health center	Minute	
		Walking distance to veterinary services	Minute	
		Walking distance to the nearest market	Minute	
		Walking distance to credit and saving institutions	Minute	
	Social Institutions or network	HHs not involved in cooperative/CBD membership.	%	[32,35,44]
		HHs who received help from others	%	
		HHs without agricultural advice or training	%	
		HHs don't use credit services.	%	
		HHs not part of debo/working together	%	

Key: HH (Household); yrs. (years); ha (hectare).

Every household head and agricultural expert in the districts were part of the study population. *Amharic*, the local language, was employed for the survey. Selected survey enumerators with appropriate skills and experience who spoke the local language were trained on how to administer the questionnaire. Before distribution, the questionnaire was reviewed, and any unclear questions were clarified. Data was collected from September 2023 to November 2023. Before the questionnaire and interviews were conducted, respondents signed consent forms. These forms are intended for respondents or research participants to indicate their agreement or refusal to participate.

Monthly time series records of gridded minimum and maximum temperature data, with a spatial resolution of 4 km × 4 km, were collected from the Ethiopian Meteorology Agency for the period from 1990 to 2018. The researcher aimed to utilize meteorological data covering 32 years. However, due to the unavailability of the necessary data covering the years 1981–2020 from the Ethiopian Meteorological Agency, the researcher was constrained to use only the available data from 1990 to 2018. Time series data representing the lowland, midland, and highland regions was extracted from blended raster data using R Studio for specific points. The data were used to analyze trends and assess the vulnerability of the local community to climate-related shocks.

Three focused group discussions (FGDs) were hosted in each sampled *kebele*, with participants 7–9 members in each meeting. A total of 45 members engaged in group discussions across all six *kebeles*. The FGDs included religious leaders, local community leaders, representatives of women, and youth. The author led each focus group, using a guided checklist. Moreover, key informant interviews were carried out with development agents, food security officials, representatives from the meteorological department, and natural resource management experts. FGDs and key informant interviews were conducted systematically, using pre-prepared questions and checklists.

### 2.4 Data analysis

The data that was gathered was analyzed using both quantitative and qualitative techniques. Eleven indicators were analyzed by the quantitative analysis, which was carried out with SPSS v25.0. Combining these methods provided a deeper understanding of livelihood vulnerability across various contexts. A thematic analysis was conducted on the qualitative data following the transcription of the audio recordings from key informant interviews and FGDs. Data management and analysis were done using XLSTAT, MS Excel, and SPSS.

#### 2.4.1 Livelihood Vulnerability Index (LVI) calculation

Assessing livelihood vulnerability helps us identify individuals who are vulnerable and determine which factors make them sensitive to climate hazards. It also shows us how climate hazards affect communal systems and resources within the framework [49]. The communities in the study area are faced with various natural hazards that impact their agricultural land, livestock, and sources of livelihood. These hazards include food insecurity, soil erosion, and drought, which make them even more vulnerable [50,51]. Three primary factors determine vulnerability: sensitivity, exposure, and adaptive capacity [52]. In essence, vulnerability has an adverse effect on adaptive capacity and a positive relationship with sensitivity and exposure [53]. The community's social identity also influences their perception of climate risks and their ability to adapt [54].

The LVI is a comprehensive, adaptable, and multidisciplinary approach that considers institutional, social, economic, environmental, and physical factors [31]. It has been used by Bedeke et al. [38] and Asfaw et al. [16] to evaluate the vulnerability of local communities to climate variability and climate change. Using the LVI-IPCC approach, eleven major components were identified, with an additional 54 sub-components under these selected components. Local conditions, literature reviews, field surveys, research area characteristics, local expert opinions, data availability, and assumptions about functional cause-effect relationships were considered when selecting the sub-components. A detailed description of the main components and sub-components is presented in Table 1.

This study follows the approach employed by Hahn et al. [31] in determining household livelihood vulnerability. Similar to how it is used to determine life expectancy, the Human Development Index (HDI) concept was used to standardize the indicator values of sensitivity, exposure, and adaptive capacity [55]. Each household head was requested to provide an impact value for each sub-component using continuous, nominal, or ordinal scales. Subsequently, the normalization of each subcomponent was computed using Equation (1) [31,36].

(1) $$\text{Inde}x_{a}=\frac{s_{r}-s_{\min}}{s_{\max}{-s}_{\min}}$$

whereas indicators expected to be inversely related to vulnerability were standardized using Equation (2):

(2) $$\text{Inde}x_{a}=\frac{s_{\max}-s_{r}}{s_{\max}-s_{\min}}$$

where index_a_ represents the standardized value of the indicator for a specific AEZ, S_r_ stands for the observed value of the indicator for the chosen AEZ, S_min_ is the minimum value, and S_max_ is the maximum value of the indicator.

After the sub-components were normalized, PCA was used to assign different weights to the indicators, thereby addressing the uncertainty associated with equal weighting due to the diversity of sub-components used [56,57]. Once each indicator was standardized, the mean of the sub-indicators was calculated using Equation (3), resulting in the determination of major component values.

(3) $$M_{a}=\sum_{i=1}^{n}\frac{\text{inde}x_{\text{ai}}}{n}$$

where Ma represents one of the eleven major components of the specified AEZ, and Index_ai_ denotes the value indexed by i within each major component. The letter n stands for the total number of indicators in each major component. Using Equation (4), the values of each of the eleven major components for each AEZ were averaged to produce the AEZ-level LVI.

(4) $$\text{LV}I_{r}=\frac{\sum_{i=1}^{11}w_{i}a_{i}}{\sum_{i=1}^{11}w_{i}}$$

whereas LVI_r_ indicates the overall index for eleven major components, each major component’s weight is denoted by *w*_*i*_, n is the number of sub-components that that comprise a particular major component group is denoted by n, and the sub-components indexed by i are denoted by a_*i*_. The LVI ranges from 0 to 1, with a lower value indicating lower vulnerability. As the LVI value increases, the vulnerability level also increases. A value of 1 on the LVI scale indicates an extremely vulnerable condition, as described by Shah et al. [58].

#### 2.4.2 LVI according to the IPCC approach

The LVI was calculated for this study using the IPCC (LVI–IPCC) framework technique. The method was developed as a substitute to calculate livelihood vulnerability in a particular area. It incorporates the vulnerability definition provided by the IPCC [59], providing a more comprehensive and effective method. Many researchers have used this technique [15,51,60]. The LVI-IPCC approach quantifies the current resilience of livelihood and health systems and communities’ capacity to develop suitable approaches in response to climate-related risks.

The LVI-IPCC framework utilizes the three indicators of vulnerability (adaptive capacity, exposure, and sensitivity) to incorporate the eleven main components. Lastly, it was combined to find the vulnerability of the individual capital of sustainable livelihood framework (SLF). Sociodemographic profiles, livelihood strategies, knowledge, wealth and income, technology, infrastructure, and social networks all combined to form adaptive capacity. The sensitivity index was composed of agricultural systems, natural resources, and water-related issues, while natural disasters and climate variability were exposure factors. Lastly, the LVI-IPCC was calculated using Equation (5) based on recommendations of Alam et al. [61], Dechassa et al. [62], and Hahn et al. [31].

(5) $$\text{LVI}-{\text{IPCC}}_{v}=\left(Ex_{V}-Ad_{V}\right)*S_{V}$$

where LVI-IPCC_V_ is the LVI for the AEZ V represented based on the IPCC vulnerability framework; E*x*_*v*_, A*d*_*v*,_ and *S* are the computed exposure, adaptive capacity, and sensitivity scores for agroecology V, respectively. The LVI-IPCC value is between −1 (least vulnerable) to 1 (most vulnerable).

## 3 Results and discussions

Two phases are used to illustrate the results of the vulnerability for three AEZs. Initially, the assessments involve individual profiles and the contributions of indicators to each profile for the three AEZs, along with an overall LVI. The extent of vulnerability for the agroecological zones is then determined using the LVI-IPCC by taking into account the exposure, sensitivity, and adaptive capacity of the climatic vulnerability index. The LVI-IPCC identifies the primary contributing factors within the exposure, sensitivity, and adaptive capacity that define the vulnerability of AEZs, while the LVI highlights the specific contributing elements.

### 3.1 Exposure

Exposure is a key factor in determining household vulnerability, reflecting the type and extent of susceptibility of agro-based livelihood systems to significant changes in climate conditions [59]. In this study, the exposure profile of households includes two sub-components: climate change and climate variability, along with natural disasters, which are represented by the 11 indicators shown in Table 2.

**Table 2:** Indexed exposure scores across the three agro-ecological zones of the study area.

Sub-component	Unit	Index Scores			Major component	Index Scores		
		Highland	Midland	Lowland		Highland	Midland	Lowland
Mean monthly maximum temperature with standard deviation (1990–2022)	^o^C	0.041	0.031	0.039	Climate Variability	0.762	0.744	0.744
Mean monthly minimum temperature with standard deviation (1990–2022)	^o^C	0.061	0.051	0.056				
Mean SD of average monthly rainfall (mm) (1990–2022)	mm	0.044	0.042	0.034				
Households who perceive increasing temperature trends	%	0.447	0.442	0.437				
HHs that believe rainfall has decreased during the rainy season	%	0.169	0.178	0.178				
HHs who perceive the occurrence of drought over 20 years.	%	0.248	0.281	0.265	Natural Disaster	0.655	0.680	0.668
HHs who perceive delayed onset and early cessation of rainfall	%	0.163	0.142	0.175				
HHs who reported crop disease/pest outbreaks over 20 years.	%	0.175	0.166	0.198				
HHs who reported livestock disease outbreaks over 20 years.	%	0.034	0.033	0.005				
HHs lack prior warning about natural disasters.	%	0.034	0.057	0.025				
Exposure LVI						0.709	0.712	0.706

#### 3.1.1 Exposure vulnerability factor: climate variability and climate change profile

The climate profile illustrates the potential risks linked to exposure to climate variability or change and consists of a set of indicators related to these climatic factors, comprising five specific indicators. The analysis shows that midland agroecology exhibits the highest level of vulnerability, with a value of 0.712, followed by the highland (0.709) and lowland (0.706). Compared to the lowland and midland agroecology, the study shows a noticeable increase in temperature and a corresponding decline in rainfall in the highlands. Therefore, the results highlight the significant vulnerability of these three agroecological zones to climate change-induced shocks.

The analysis of this study shows that changes in rainfall patterns emerge as the primary climate-related stressor in the midlands. Similar studies have shown a correlation between a higher prevalence of climate-related risks and increased vulnerability to exposure [63,64]. Studies conducted by various scholars [12,37,39] consistently highlight varying levels of exposure to climate-related challenges among households in different agroecological settings.

In this study, households were asked about changes in rainfall and temperature over the past two decades. An analysis of household perceptions of climate variability revealed that the majority of respondents across the three AEZs reported rising temperatures and decreasing rainfall over this period. However, the findings varied among AEZs. Households in lowland and midland AEZs reported a greater decrease in rainfall (88 %) compared to those in highland agroecology (84.5 %).

Conversely, over 83 % of households across all AEZs reported a temperature increase. The highland zone had the highest percentage (85 %) of households perceiving an increase in temperature, followed by the midland (84 %) and lowland (83 %) zones. This indicates limited variation in household perceptions of temperature increase across the three AEZs. Earlier studies have confirmed that unpredictable precipitation patterns lead to reduced agricultural output, resulting in food shortages and higher vulnerability for families in East African nations [65].

The results of focused group discussions further support this finding. These results align with earlier studies conducted in other parts of Ethiopia [37,39,62], which indicate that farmers have noticed an increasing trend in temperature and a decrease in rainfall. In addition, households have reported a higher occurrence of extreme climate events, such as off-seasonal rainfall, which serve as important indicators of changes across the three agroecology. When it comes to drought occurrence, a higher percentage of households from the midland AEZ (85 %) supposed that the incidence of droughts had increased compared to those in the lowland and highland agroecology.

The Mann-Kendall (MK) trend test and Sen's slope estimator were used to assess the trend and magnitude of annual rainfall as well as the minimum and maximum temperatures of the three AEZs. The study was conducted at a 5 % significance level with a 95 % confidence interval, covering the period from 1990 to 2022. The mean annual rainfall in the area ranges from 844.36 mm to 1186.9 mm.

The analysis of meteorological records indicated statistically insignificant increasing trends in annual rainfall across the three AEZs. The magnitude of the trend in annual rainfall varies spatially from 2.028 to 2.917 mm per year, being lower in the lowland and higher in the highland AEZs of the study area. Similar findings on the increasing trend in mean annual rainfall have been reported in previous studies [66,67]. In all AEZs, an increasing trend in mean annual minimum temperature was observed; however, this increase was statistically insignificant. According to Sen's slope analysis, the rate of increment in minimum temperature in the highland, midland, and lowland AEZs was 0.009 °C, 0.007 °C, and 0.008 °C per year, respectively, over the past two decades. Likewise, negligible upward trends in the maximum temperature were also noted in each of the three AEZs. In the highland, midland, and lowland AEZs, the maximum temperature increased by 0.009 °C, 0.011 °C, and 0.009 °C per year, respectively. The findings show that the midland AEZ has a higher annual rate of increase in maximum temperature than the highland and lowland regions. The detailed results are available in Table 3.

**Table 3:** Mann-Kendall trend test of rainfall, temperature minimum, and temperature maximum across three AEZs.

AEZ	Rainfall				Temperature minimum				Temperature maximum			
	Z-value	Sen’s slope	P-value	Tau	Z-value	Sen’s slope	P-value	Tau	Z-value	Sen’s slope	P-value	Tau
Highland	0.511	2.92	0.371	0.064	1.863	0.009	0.000	0.227	1.162	0.009	0.121	0.143
Midland	1.409	2.87	0.005	0.022	1.936	0.007	0.000	0.238	1.564	0.011	0.026	0.193
Lowland	0.387	2.03	0.484	0.049	1.719	0.008	0.000	0.212	1.038	0.009	0.169	0.128

Contrary to the results of meteorological data, households perceive a decrease in rainfall. Participants in the focus groups reported fluctuations in rainfall, temperature increases across all agroecological zones, recurring dry periods, and subsequent decreases in crop yields. Key informants consistently reiterated these observations.

According to a study conducted by the National Meteorological Agency (NMA) of Ethiopia, the average annual temperature has increased by 0.37 °C per decade. The study also reported an additional rise of 0.1 °C in the mean maximum temperature. Over the previous 50 years, the nation has also experienced notable variations in rainfall [68]. It is important to note, however, that statistical significance in changes in rainfall is not evident. Both meteorological records and information provided by respondents confirm the fluctuation of rainfall, which leads to shifts in the agricultural calendar. Thus, while various models consistently suggest a rising temperature trend, precipitation patterns show both increasing and decreasing trends, depending on the specific model employed [68].

Over the past 55 years, there have been periods of drought and famine due to dry conditions, and periods of wet conditions [69]. Furthermore, there is a non-significant downward trend in the actual rainfall records during the past 20 years. The rates are 2.03 mm/year, 2.87 mm/year, and 2.92 mm/year in lowland, midland, and highland agroecology, respectively. Specifically, the analysis of rainfall indicates a decrease of 2.60 mm in mean annual rainfall during recent years. Esayas et al. [70] reported similar findings, noting an upward trend in average annual temperature and a general decline in precipitation between 1981 and 2011.

#### 3.1.2 Exposure vulnerability factor: Natural disaster

The factors that lead to natural disasters, such as crop failure, animal diseases, drought intensity, length, and frequency, are included in the natural disaster profile. Households were asked to identify the contributing factors to natural disasters that they had faced or experienced over the past twenty years. The majority of households reported drought, crop failure, and livestock disease were the most common factors contributing to natural disasters. The occurrence of these factors varied significantly among different agroecology.

The natural disaster risk scores indicate that all agroecology exhibited elevated scores, with values of 0.655, 0.680, and 0.668 for highland, midland, and lowland zones, respectively (Table 2). This implies that all agroecology possesses a considerable susceptibility to natural disasters. The higher exposure index in the midlands can be attributed to a higher incidence of crop diseases and livestock/animal damage caused by natural disasters. This provides valuable information on the areas that require prioritization in terms of intervention for managing climate change-induced risks in agricultural and livelihood environments. This provides valuable insights into the areas that require prioritization in terms of intervention for managing climate change-induced risks in agricultural and livelihood environments.

The overall impact of disaster risk becomes evident when describing the exposure profile of households. Over the past two decades, a majority of households consistently faced frequent drought, livestock diseases, and crop failures. Earlier studies conducted in different regions of Ethiopia [3,39,71] have shown that droughts occur more frequently in lowland areas than in midland and highland regions. Furthermore, these studies have also highlighted the associated risks of droughts in these areas. The majority of households said that climate-related threats, including droughts, frost, erratic rainfall, invasive weeds and pests, and diseases, had become more frequent in all agroecological zones. These factors, which are impacted by climate change and unpredictability, have intensified and are having a major effect on crop productivity and people's quality of life.

In the lowland AEZ, the primary climate-related stressors affecting crop production and livelihoods are erratic rainfall patterns, accounting for 80 % of the cases, and drought, accounting for 49.3 % of the cases. Conversely, households in the midland AEZ stated that frost and snow are the primary climate-related factors influencing their livelihoods.

The findings align with previous research indicating an increase in climate change and variability, leading to events of excessive and deficient rainfall, as well as rising temperatures. These factors may put additional demand on medical facilities as well as food and water supplies [72]. The capacity to offer ecological services in Ethiopia is limited by environmental challenges such as land degradation, soil erosion, and climate-related hazards like recurrent droughts, floods, heavy rains, frost, or rising temperatures. This makes it difficult to obtain and make use of resources for livelihood [68]. Moreover, researchers contend that households may be more vulnerable to climate disasters as a result of repeated environmental and socioeconomic shocks [73].

Participants from all three agroecology mentioned a rise in the frequency of frost and storms. Individuals from each agroecology also pointed out that rainfall has become less predictable in terms of how often it occurs, when it occurs, and how intense it is. During focus group discussions, it was noted that rainfall used to be common from June to September, particularly in lowland areas. However, nowadays, it occurs irregularly due to limited rainfall and the early drying up of water reserves caused by high evaporation. As a result, participants have developed a better awareness of climate change, which has made them feel less vulnerable to climate-related shocks. The way a household views climate change is crucial to managing its effects [74]. A lack of understanding about how the environment is changing can increase respondents' exposure to risks. Conversely, Altieri et al. [75] argued that in areas with less observed drought, such as highlands and lowlands, households are unlikely to perceive it as a significant occurrence.

### 3.2 Sensitivity vulnerability factor

The evaluation of household sensitivity associated with climate variability had two main components: water and agriculture systems and natural resources. This includes adaptations in both natural ecosystems and regulated systems, such as agriculture. The sensitivity of agroecological systems was assessed by examining variations in ecosystem and agricultural characteristics. Six indicators were employed for each agroecology, as shown in Table 4. According to the sensitivity analysis results, the lowland agroecology showed the highest sensitivity (0.671), followed by the highland (0.572) and midland (0.533) agroecologies.

**Table 4:** Sensitivity analysis index scores for each of the three agroecological zones in the study area.

Indicators	Sub-component		Index Scores			Index Scores		
		Units	highland	midland	lowland	highland	midland	lowland
Agriculture system and natural resource	Crop diversification index	1/#crops	0.017	0.012	0.032	0.653	0.634	0.676
	Households who perceive rapid natural vegetation conversions	%	0.444	0.439	0.459			
	HHs who have fertile farmland	%	0.058	0.077	0.074			
	HHs who have land tenure insecurity feeling	%	0.062	0.054	0.074			
	HHs who perceive rapid land degradation	%	0.021	0.003	0.004			
	Landholding size	Ha	0.051	0.050	0.033			
Water	HHs who reported water conflicts	%	0.062	0.001	0.077	0.449	0.382	0.664
	HHs who use a natural water source	%	0.139	0.114	0.193			
	Average distance to a water source by foot	Minute	0.116	0.116	0.186			
	HHs who haven't a consistent water supply	%	0.131	0.151	0.209			

#### 3.2.1 Agriculture system and natural resource

In contrast to highland and midland regions, lowland agroecology is more sensitive when considering the agricultural system and natural resources. The sensitivity values for lowland, highland, and midland agroecology are 0.676, 0.653, and 0.634, respectively. To determine the vulnerability profiles of the agriculture system and natural resources, six indicators were considered: crop diversification index, households' perception of natural vegetation conversions, households with fertile farmland, insecurity in land tenure, households perceiving fast land degradation, and households' land holding size. In terms of rapid natural vegetation conversions alone, households are more sensitive than the other subcomponents, with values of 0.444 for highland, 0.439 for midland, and 0.459 for lowland areas.

As the local community in the study area relies on a subsistence and mixed farming system, the significance of being environmentally aware of ecosystem services becomes crucial. Table 4 provides a summary of eleven indicators used to assess the sensitivity of households to climate change and variability. Factors such as low crop diversification, households with limited access to fertile farmland, along issues like fast land degradation and small landholding sizes, have all negatively impacted the agriculture system and natural resources.

#### 3.2.2 Water

The increasing incidence of severe events coupled with ongoing pressure on water supply and quality indicates the increasing threat posed by climate change to water distribution systems [76]. The LVI showed that the lowland had a greater sensitivity value of 0.664 in the water resource component, whereas the midland and highland had weighted average scores of 0.382 and 0.449, respectively.

Numerous households in lowland areas have reported natural springs and other water supplies frequently dry up over the dry season. The accessibility of water in these sources varies as well. This is in line with studies that demonstrate lowland agroecology regions are more vulnerable to water-related issues compared to midland and highland regions [16,41]. Vulnerability in rural regions is significantly influenced by dependence on water supplies for agriculture and the condition of existing infrastructure in the sector [77]. The analysis of water vulnerability profiles considered four indicators: conflicts arising from water scarcity, lack of access to clean water, distance households must travel to obtain the main water source and inconsistency of water supply.

In lowland areas, 61.3 % of households lack adequate access to clean water for domestic use, a higher percentage compared to households in highland (44.3 %) and midland (36.2 %) AEZs. Key informant interviews and focus group talks further revealed the severity of the issue during the dry season, especially in lowland areas. According to previous studies [3,71], the challenges of acquiring water for daily household activities significantly the vulnerability of lowland communities experiencing severe water shortages. In terms of consistent water supply, the lowland agroecology scored the highest, with a mean weighted score of 0.208, followed by the midland and highland AEZs, with mean weighted scores of 0.150 and 0.131, respectively.

In terms of the distance to the nearest water source for home consumption, the lowland agroecology had an average time of 0.75 h, exceeding that of the midland (0.34 h) and highland (0.31 h) agroecology.

As a vulnerability indicator, the distance to water sources implies that the more time family members spend fetching water, the less time they have for other income-generating activities. This burden falls heavily on women and children, affecting school attendance and exposing livestock to physical degradation and increased susceptibility to pests and diseases. Since rainwater can be a reliable source for both irrigation and drinking, it is essential to encourage local populations to adopt rainwater collection systems.

### 3.3 Adaptive capacity

**Fig. 2:**
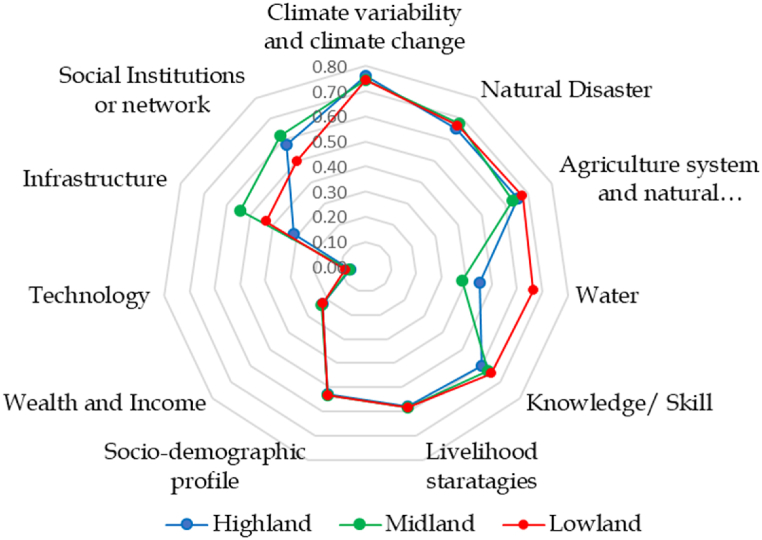
The spider diagram depicts the LVI of the principal components.

The relationship between exposure, sensitivity, and vulnerability implies that areas with higher exposure and sensitivity levels are anticipated to face greater vulnerability to climate-related effects. However, the situation takes a different turn concerning adaptive capacity. It is expected that individuals or systems with higher adaptive capacity will be less susceptible to climate change and variation. Adaptive capacity refers to the ability to handle change and adapt to changing circumstances. Numerous socioeconomic factors, such as affluence, the adoption of agricultural technology, the development of infrastructure, and social capital traits, all have an impact on adaptive capacity [71]. Seven sub-components were used in this study to create the index of households' adaptive capacity: social networks, technology, livelihood strategies, wealth and income, knowledge and skills, and sociodemographic profile (Fig. 2).

In this study, the adaptive capacity index for households was constructed by incorporating seven sub-components: socio-demographic profile, livelihood strategies, knowledge and skills, wealth and income, technology, infrastructure, and social networks. The results showed how each indicator contributed to the adaptive capacity of the three agroecological zones in adapting to and coping with climate-related hazards. The overall adaptive capacity index values for the highland, midland, and lowland areas were 0.415, 0.472, and 0.438, respectively, suggesting that the midland exhibited the highest adaptive capacity (indicating lower vulnerability), followed by the lowland. In contrast, the highland demonstrated the lowest adaptive capacity.

#### 3.3.1 Socio-demographic profile

The socio-demographic profile analysis includes six indicators: education level, dependency ratio, farming experience, age of household heads, and female as household head. Household characteristics which include family size, dependence ratio, educational attainment, and sex of the household head have a substantial effect on a community's ability to adapt and respond to climate-induced consequences [22,74,78]. The findings indicate that in terms of sociodemographic profile, the lowland agroecology demonstrated a comparatively high adaptive capacity, as evidenced by its weighted average value of 0.533. This was followed by the midland and highland, with values of 0.533 and 0.529, respectively. Previous studies have extensively examined the association between farmers' socioeconomic and demographic characteristics, their vulnerability to climate change and the adaptation techniques they used [79,80].

A hypothesis suggests that households headed by men who have a low dependency ratio and a family size that is not higher than the national average would be less vulnerable to climate change. The adoption of climate-related risks is mostly influenced by the gender of the head of the household. Because of cultural norms that limit women's access to agricultural extension services, women-headed households are more vulnerable than men.

The midland agroecology demonstrated the maximum level of adaptive capacity when considering only the number of female-headed households, with a mean score of 0.179. It was closely followed by the highland with a value of 0.174 and the lowland with a score of 0.171. The lowland had a slightly higher proportion of female-headed households (22.5 %) compared to the midland (18.5 %) and highland (21 %) areas. Female-headed households faced greater vulnerability to adverse climate conditions due to gender differences [16].

In terms of the dependency ratio, a small variation was noticed across the three agro-ecologies. The results revealed that the dependency ratio was higher in the lowland (54.6) compared to the highland (53.4) and midland (49.4). This implies that the productive age category of family members in the midland agroecology was better than in the lowland and highland regions. In other words, there were more capable working family members available to support dependents in the midland compared to the highland and lowland regions. This discrepancy may make the lowlands and highlands more susceptible to climate-related risks compared to the midland region.

A higher dependency ratio makes households more vulnerable to the effects of climate variability and change. This is because it diminishes their capacity to afford food prices [81]. Moreover, the results indicate that lowlands have higher access to productive employment opportunities, which can address the economic and social needs of families, compared to highland and midland regions. Households reported facing challenges, particularly during climate fluctuations, due to a significant number of dependent family members. The underlying vulnerability in this context arises from the fact that households with a greater number of dependents are more likely to face vulnerability. This is because a larger proportion of their resources are allocated to dependents who contribute minimally to household welfare [74]. The analysis of this study aligns with earlier studies, suggesting that having a larger working-age family could serve as a form of insurance. It contributes to the development of human capital that enhances resilience in livelihoods through access to employment, remittance earnings, and risk management [82].

The highland area demonstrated the highest degree of adaptive capacity when the educational qualifications of household heads were examined, with a mean score of 0.073. This was followed by the midland and lowland areas, which had a mean weighted score of 0.067 and 0.064, respectively. The variation is likely due to a greater proportion of households lacking formal education in the lowlands. According to the results, more than 60 % of respondents in each of the three studied AEZs lacked basic literacy skills, which exacerbates their vulnerability to the adverse effects of climate change.

As per the focus group discussion, low awareness and illiteracy emerge as primary factors contributing to farmers' inadequate capacity to address climate-related risks. Farmers who have received education and training are more capable of adopting new technologies, thus enhancing their ability to build resilience against the challenges brought by climate change and variability [83]. Other studies have also highlighted the significant role of the household head's educational level in household decision-making, planning, and overall well-being [45,84]. Educated households can navigate the challenges posed by climate change and explore alternative options [31]. Education also helps households better understand extension services and take advantage of many opportunities in emergencies [84,85]. Conversely, households with low literacy levels encounter obstacles in accessing climate change information, participating in training programs, and diversifying their skills, rendering them particularly susceptible to the influences of climate-related shocks.

Farming experience is another indicator within the socio-demographic profile, which offers a chance to reduce susceptibility to the negative consequences of climate change through adaptable planting practices, crop selection, and improved farm management techniques [86]. The majority of households have considerable experience in farming, with slightly over half of the respondents across all agroecology reporting 18–35 years of farming experience. Notably, in midland and highland agroecology, this proportion is higher than in the lowland. The study reveals that households in the midland, on average, possess more years of farming experience (26.1 years) compared to those in the highland (26 years) and lowland (24.2 years) agroecology. As a result, households in the midland areas were less vulnerable because they were better equipped to adjust to the possible impacts of climate change.

Farming experience of households highlights that farmers in the midland agroecological zone possess relatively more experience than their counterparts in the lowlands and highlands. This difference may be attributed to a higher percentage of households with limited farming experience in the lowland and highland areas. Farming experience plays a vital role in enhancing the sustainability of farmers' lives through improvements in farming practices. Bryan et al. [80,87] highlighted the necessity for substantial government involvement and leadership in planning to effectively adapt and implement best practices at a small scale. In light of the low levels in the study area, it is crucial to actively promote farmer-to-farmer experience exchange to improve the adoption of agricultural technologies. The results also indicate that the mean family size was 5.0 in the highland, 4.8 in the midland, and 4.5 in the lowland. A study conducted by Zeleke et al. [35] in North Wello, Ethiopia revealed similar findings. The possibility of having more dependency rises with family size, increasing the household's susceptibility to climate shocks [33].

#### 3.3.2 Livelihood strategies

A livelihood strategy refers to a plan adopted by households to ensure survival, build wealth, manage risks, and alleviate poverty. Households in the three agroecological zones employ diversified livelihood strategies, including diversification and coping strategies, improved livestock breeding and technology, the application of fertilizers and compost or manure, reliance on rainfed agriculture for income, coping with food insecurity, and participation in non-farm activities.

The majority of households in the highlands (95 %), midlands (96 %), and lowlands (96.5 %) agroecologies depend solely on agriculture as their primary source of income. However, this reliance makes them highly sensitive due to the climate-sensitive nature of the agricultural sector [48]. The findings of this study show that the level of sensitivity in the highlands was higher compared to the lowlands and midlands. Households that depend only on agriculture are thought to be more susceptible to climate change than those that have other sources of income [31]. Households in the study areas rely heavily on agriculture as their main source of income. As noted by Abdi et al. [88], this situation is attributed to the absence of alternate sources of income in the area. The findings of this study are in line with those of a study carried out by Mekonnen et al. [89] in the Central Rift Valley, Ethiopia.

Formal education increases a household's adaptive capacity by improving its ability to recognize challenges. Consequently, it enables the household to seek feasible solutions from appropriate sources [90]. The findings indicate that about 67 % of household heads in the highlands, 69.6 % in the midlands, and 71 % in the lowlands have not attended formal school. A small number of households in all AEZs have completed or attended primary and secondary school. This suggests that respondents in the highlands were more likely to attend school due to better conditions and educational facilities compared to those in the lowland and midland areas, indicating lower education coverage in the latter regions. Similar studies have also shown that farmers with low literacy levels are more vulnerable to climate-related stress because they have less access to information, particularly from written materials [91,92].

Most households in the study areas had small farms, with average land sizes of 0.9 ha, 1.0 ha, and 1.7 ha in the highland, midland, and lowland areas, respectively. The average landholding size of the sampled households in this study is 1.12 ha, which is slightly higher than the national average. A national land use survey indicates that the mean household landholding size in Ethiopia is 0.92 ha, with an average cropland area of 0.78 ha [93]. The larger landholding size owned by farmers may contribute to a higher natural component score. In the study area, approximately 75 % of the respondents have landholdings of 1 ha or less. Given that land is a crucial asset and a primary source of rural livelihoods, having a smaller landholding size in the context of a changing climate suggests weaker adaptive capacity and increased vulnerability. It is imperative to note that in Ethiopia, the size of landholdings and yearly household income are strongly correlated [94,95].

The results indicate that the average TLUs for the lowland, midland, and highland agroecologies were 4.1, 3.1, and 4.1 TLUs, respectively. The findings show that households in the lowlands held more land compared to their counterparts in the midland and midland regions.

A notable distinction existed among the three areas concerning farmers' participation in *debo* (working together) and in cooperative or community-based development centers. According to the study, most households across three agroecological settings used both artificial fertilizer and compost/manure, with a fairly uniform distribution.

Regardless of the agroecological zone, households typically spend more than an hour traveling to access market centers, health facilities, and credit and savings institutions. However, the journey to reach extension services took less than an hour only in the highland and midland areas. The susceptibility of households to climate change is primarily attributed to weaknesses in wealth and income. The findings show that most respondents across the three districts were between the ages of 30 and 49 years.

The composite LVI and major components for each agroecology are shown in Table 5. The findings showed that for each of the three AEZs, the livelihood vulnerability indices for the main indicators ranged from 0.498 to 0.531. The LVI indicates that lowland households are somewhat more vulnerable to climate change than midland and highland households (Table 5). By comparing the various indices across the three agroecologies, this analysis highlights potential variations in livelihood vulnerability across different AEZs.

**Table 5:** The indexed values for indicators, components, and the overall LVI across the highland, midland, and lowland AEZs.

Component	Major Components	No of indicators	Indexed values		
			Highland	Midland	Lowland
Exposure	Climate variability and climate change	5	0.762	0.744	0.744
	Natural Disaster	5	0.655	0.680	0.668
	**Contributing factor**	**10**	**0.709**	**0.712**	**0.706**
Sensitivity	Agriculture system and natural resource	6	0.653	0.634	0.676
	Water	4	0.449	0.382	0.664
	**Contributing factor**	**10**	**0.572**	**0.533**	**0.671**
Adaptive Capacity	Knowledge/Skill	3	0.601	0.633	0.651
	Livelihood strategies	6	0.580	0.581	0.584
	Socio-demographic profile	6	0.529	0.532	0.533
	Wealth and Income	4	0.224	0.231	0.224
	Technology	4	0.062	0.065	0.082
	Infrastructure	7	0.314	0.542	0.432
	Social Institutions or network	4	0.578	0.619	0.500
	**Contributing factor**	**34**	**0.415**	**0.472**	**0.438**
		LVI	**0.498**	**0.528**	**0.531**
		LVI-IPCC	**0.168**	**0.128**	**0.180**

#### 3.3.3 Knowledge and skill

In this study, the knowledge and skill component consists of three sub-components: the percentage of households that received training on climate change adaptation, the percentage of households that did not receive health extension services, and farm management practices.

The analysis reveals that the knowledge and skill component received the highest weighted mean score in the lowland (0.65), followed by the midland (0.63) and highland (0.60). Although the lowland had a higher percentage of households (52 %) receiving training to cope with climate change compared to the highland (45 %), the other two contributing factors explain the lowland's greater vulnerability. Compared to the lowland agroecology (62.7 %), results show that a higher proportion of midland household heads (74.4 %) did not receive farm management training. In contrast, the lowland had a higher percentage of households (82.0 %) without access to health extension services, compared to the midland (75 %) and highland (65 %). Smallholder farmers are highly vulnerable to climate-induced shocks due to their heavy reliance on climate-sensitive sectors. Additionally, poor land use practices further exacerbate this vulnerability [33,71].

#### 3.3.4 Agricultural technology

Four indicators were taken into account to assess agricultural technology vulnerability: households using better-quality seeds, households using insecticides and pesticides, households practicing irrigation, and households with radio access. According to the overall agricultural technology vulnerability score, all agroecologies exhibited low adaptive capacity. However, the lowland area demonstrated a higher adaptive capacity, with a mean score of 0.08, compared to the midland and highland, which had average weighted values of 0.065 and 0.062, respectively.

#### 3.3.5 Wealth and income

In the wealth and income major component, four indicators were identified: TLUs, households without a house built with wood and corrugated iron, income from crop production, and households' non-farm income. The average vulnerability scores for households in the midland agroecology were 0.22, 0.23, and 0.22, respectively. These scores indicate that households in the midland agroecology are significantly more vulnerable to wealth and income challenges compared to highland and lowland. Therefore, the impact of climate change on wealth and income is determined by the level of exposure and vulnerability to these shocks [45]. However, several studies have revealed that lowland agroecology is more vulnerable regarding wealth and income compared to other agroecologies.

#### 3.3.6 Infrastructure

The overall availability of basic infrastructure in the three agroecological areas is limited, as shown in Table 6, highlighting their vulnerability to climate change-induced shocks. Notably, the midland agroecology demonstrated comparatively better access to basic infrastructure, with a score of 0.542, followed by the lowland (0.432) and highland (0.314).

**Table 6:** Average time spent to access basic infrastructure services from home and indicators.

Major Component	Indicators	Unit	Agro-ecology			Weighted average score		
			Highland	Midland	Lowland	Highland	Midland	Lowland
Infrastructure	Walking distance to main weather roads	Minute	0.007	0.041	0.037	0.314	0.542	0.432
	School distance covered on foot.	Minute	0.007	0.039	0.037			
	Extension services distance covered on foot	Minute	0.006	0.042	0.030			
	Health center distance covered on foot	Minute	0.048	0.075	0.041			
	Walking distance to veterinary services	Minute	0.040	0.069	0.035			
	Nearest market distance covered on foot	Minute	0.137	0.171	0.165			
	Walking distance to credit and saving institutions	Minute	0.068	0.105	0.087			

#### 3.3.7 Social institutions or network

The social network component consists of four sub-components: cooperative/CBD membership involvement, receiving agricultural advice/training, accessing credit services, and participating in debo/working together. In terms of the LVI related to the social network, the midland agroecology exhibited a value of 0.62, indicating a higher level of vulnerability compared to the lowland (0.50) and highland (0.58) areas. Well-established and well-coordinated social networks are essential for lowering households’ and communities’ vulnerability to climate change [31].

The livelihood vulnerability indicators for the eleven essential components are presented using a spider diagram (Fig. 2). The scales in the diagram have intervals of 0.1, with zero at the center representing the least vulnerability and 0.8 at the outer edge representing the greatest vulnerability. The findings from the spider diagram align with the LVI analysis of the respective components. The lowland area stands out for its high sensitivity but low adaptive capacity compared to the midland and highland regions. The diagram clearly shows that the LVI score for each of the three agroecologies is significantly influenced by climate variability and change, the components of the agricultural system, natural resources, and natural hazards. In particular, the primary contributor to the lowland agroecological zone's vulnerability is the water component. In contrast, infrastructure and social institutions or network components appear to be the most influential factors for LVI calculations in the midland area.

### 3.4 Evaluation of household Livelihood Vulnerability Index

The results of the LVI-IPCC showed that the three agroecologies exhibited varying degrees of vulnerability, with scores ranging from −1 to +1 [45]. According to the LVI-IPCC computations, exposure with adaptive capacity produced positive vulnerability values, whereas lower values produced negative scores, which ranged from −1 (showing the least vulnerable) to +1 (representing the most vulnerable). The results of the study revealed that the LVI-IPCC was highest in the lowland area (value of 0.198), while the values in the highland and midland zones were, respectively, 0.171 and 0.131 (Table 7). To compute the LVI-IPCC, eleven main components were divided into three groups: exposure, sensitivity, and adaptive capacity.

**Table 7:** Each of the three AEZs' contributing factors to the LVI-IPCC.

LVI-IPCC contributing factors	Agro-ecology		
	Highland	Midland	Lowland
Exposure	0.709	0.712	0.725
Sensitivity	0.572	0.533	0.671
Adaptive capacity	0.409	0.466	0.430

LVI-IPCC value	0.171	0.131	0.198

**Fig. 3:**
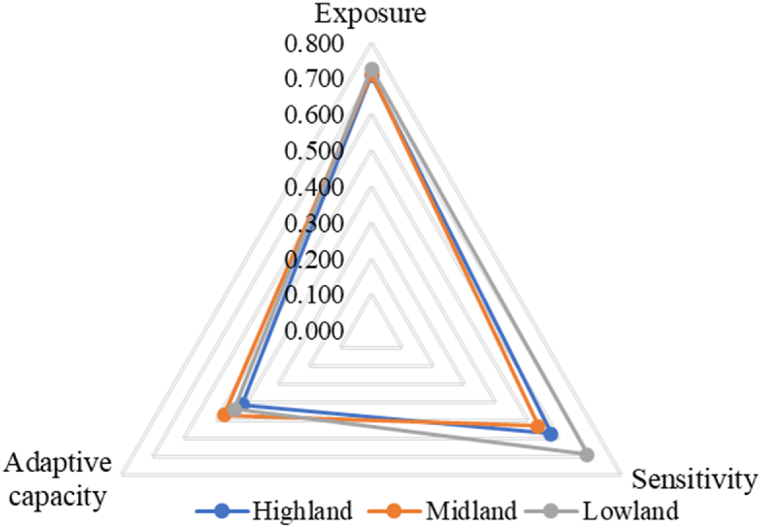
The LVI-IPCC vulnerability triangle diagram.

Fig. 3 illustrates the vulnerability triangle and the factors contributing to the LVI-IPCC. In all three AEZs, households were vulnerable to the negative effects of climate change due to increased exposure, sensitivity, and limited adaptive capacity. According to the diagram, the lowland area had greater exposure (0.712) to the effects of climate change than the midland and highland regions. The lowland area was also more sensitive to climate change (0.671) compared to the highlands, regardless of having a better adaptive capacity (0.430).

## 4 Conclusion and recommendations

Examining livelihood vulnerability is key to identifying at-risk communities and understanding the causes of their susceptibility. This study aids in designing tailored adaptation interventions, as households in the area exhibit different levels of susceptibility to climate change based on their adaptive capacity. The LVI result indicated that the lowland region is more vulnerable to climate variability and change than the midland and highland regions. This is confirmed by the LVI-IPCC analysis, which shows that the lowland's exposure exceeds its potential for adaptation. The lowland is still quite vulnerable to the consequences of climate change, even if it has a higher adaptive capacity for adaptation than the highland. In contrast, the midland region shows less vulnerability due to lower exposure and better adaptive capacity.

Households in the study region demonstrate low adaptive capacity due to limited adoption of agricultural technologies, poor infrastructure, weak social networks, high socio-demographic vulnerability, and low wealth and income. These factors, particularly in the lowland area, increase vulnerability to climate change and hinder adaptation, leading to lower crop yields, compromised food security, and rising poverty.

The results provide policymakers with a starting point for developing plans that support sustainable practices by balancing environmental preservation with agricultural demands. Temperature and precipitation data should be thoroughly analyzed to comprehend climate variability and how it affects household livelihoods. This study focuses on current conditions, without making medium- or long-term predictions due to data limitations, and does not address external factors that influence long-term outcomes. Repeating similar studies over time could reveal how exposure, adaptive capacity, and sensitivity evolve in response to adaptation measures, enhancing our understanding of their impact on community resilience and informing future strategies. While reducing farmers' sensitivity to climate change on a small scale is challenging, improving the adaptive capacity of subsistence households dependent on rainfall can strengthen their resilience to both current and future climate variability.

The study area is one of the most climate-sensitive regions in Ethiopia, requiring special attention from policymakers. To reduce household vulnerability and enhance their ability to adapt, a range of strategies should be promoted. These include providing access to rural microfinance services, ensuring the timely delivery of early warnings and information, implementing effective soil and water management practices, and promoting diversified non-farm income sources. Encouraging households to engage in a variety of agricultural activities, such as poultry production, and investing in training and skill development are also crucial. Additional measures, including rainwater harvesting, the use of improved seeds, and pest control, are vital for enhancing household resilience.

The study results show that the lowland area is more vulnerable to climate change compared to the midland and highland regions. The LVI-IPCC scores rank the lowland as the highest vulnerable (0.180), while the highland has the lowest vulnerability (0.128). The varying index values across agroecological zones highlight the need for tailored adaptation strategies to improve community resilience.

Establishing farmer self-help groups is essential for managing risks and accessing key resources, such as agricultural inputs, loans, and market information. These initiatives should be tailored to local needs and implemented sustainably, with adequate resources to ensure long-term success and adaptability. Policymakers should collaborate with experts and local communities to develop adaptation plans tailored to each agroecological zone. The lowland region requires specific adaptation strategies to enhance resilience due to its high vulnerability. Assessing livelihood vulnerability to climate hazards is crucial for developing long-term plans that strengthen resilience.

## CRediT authorship contribution statement

**Tatek Belay:** Writing – review & editing, Writing – original draft, Methodology, Funding acquisition, Formal analysis. **Tadele Melese Lebeza:** Writing – review & editing.

## Data availability

The data used for this study are available from the corresponding author upon reasonable request.

## Ethics approval

Ethics approval was obtained from Debre Tabor University's research ethics guidelines. Households actively participated in the study through key informant interviews, focus groups, and surveys. The Institutional Review Board (IRB) at Debre Tabor University approved the research questions, which were thoroughly reviewed to ensure compliance with ethical standards. Ethical clearance was granted following an application to 10.13039/501100022150Debre Tabor University and approval from the IRB (Ref No. DTU/123/23). Permission was granted by the IRB committee to use the data generated for the research conducted between September 2023 and November 2023.

## Participants’ consent

Before the interviews and surveys were conducted during data collection, respondents signed consent forms. These forms are intended for respondents or research participants, to indicate their agreement or refusal to participate. If they consent to participate, they are kindly asked to sign the attendance sheet provided.

## Funding statement

10.13039/100025452The International Foundation for Science (IFS), Stockholm, Sweden, provided funding for this study to the first author (Grant No. IFS Grant I2-W-6250-2, 2023).

## Declaration of competing interest

The authors declare that they have no known competing financial interests or personal relationships that could have appeared to influence the work reported in this paper.
