# Supplementary material for: Livelihood vulnerability of rural households to climate variability and change: An agroecological system-based approach in northwestern Ethiopia
Source: Heliyon. 2024 Nov 20;10(23):e40570. doi: 10.1016/j.heliyon.2024.e40570 (PMC11625134; doi:10.1016/j.heliyon.2024.e40570)
Supplement: Multimedia component 1 [file mmc1.pdf]

DEBRE TABOR UNIVERSITY

DEPARTMENT OF GEOGRAPHY AND ENVIRONMENTAL STUDIES

ANNEX 1: STRUCTURED SURVEY QUESTIONNAIRE FOR HOUSEHOLD HEADS

Dear respondent, the aim of this interview is for academic purposes only, and the information that you will provide is strictly confidential and will not be used for any other purposes. This questionnaire is prepared to assess Livelihood vulnerability of rural households to climate variability and change: An agro-ecological system-based approach in Northwestern Ethiopia. Therefore, be free to share everything you know about what I will ask you for the successful completion of this study. Your genuine response will contribute a lot to the research output and your response is confidential.

Direction: for the closed ended questions, put "√" mark as per your choice, and for open ended questions provide your answer on the space provided. Lastly, I thank you for your time and cooperation.

General Information

1. Name of District: _____
2. Name of *Kebele:* ______
3. Village: _____________
4. Household Code: ______
5. Name of Interviewer ______

A) Socio-demographic Profile of the Respondent

1. Age of household head ________________
2. Age of the household head in years: _________
3. Sex of household head: 1= Male, 2=Female
4. Marital Status 1=Married, 2= Single, 3= Divorced, 4= Widowed
5. Religion: 1= Christian orthodox, 2= Muslim, 3= Protestant, 4= Others (specify) __________
6. Educational level: 1. Can't Read and write 2. Read and write 3. Primary (1-8) 4. Secondary (9-12) 5. Tertiary (certificate and above)
7. Size of a family (*list the age and sex of every person who eats and sleeps in this house*?)

No	Age	***Sex (in number)***		
		Male	Female	Total
7.1	<14			
7.2	15-65			
6.3	>65 year			

B) SOCIO-ECONOMIC, LAND, AND RELATED CHARACTERISTICS

8. Do you have your own land? 1. Yes 2. No
9. If your answer is yes, how many hectare (*Timad*)_____________
10. What is your primary occupation? 1) Farming 2) Employment 3) Own off business
11. What do you think the crop production per hectare from year to year in the area?

    1=Increasing, 2=Decreasing, 3= Remain the Same
12. If decreasing, what are the reasons for any decrease of your cultivated land productivity

    1= Rainfall variability/shortage, 2=Lack of timely input supply, 3= Lack of oxen, 4= Land degradation, 5=Pests and Crop diseases, 6= Other, please specify__________
13. If productivity is increasing, what are the reasons for any increase in your cultivated land productivity? ____
14. What is your primary occupation? 1) Farming 2) Employment 3) Own off business
15. Your answer is farming, how long you have been practicing farming? (i.e., farming experience in years) _____________
16. What do you feel about the land tenure system? Do you feel secure planting permanent crops and implementing adaptation techniques? 1. Yes 2. No

C) PERCEPTIONS OF CLIMATE VARIABILITY, CLIMATE VARIABILITY AND NATURAL DISASTER

17. Have you heard about "climate change" before?	1= Yes, 0= No, 3= Not sure
18. Do you perceive that there is climate change in your area?	1= Yes, 0= No,
19. What has been the trend of temperature for the past 20 years in your locality?	1=Increase, 2=Decrease, 3= Not change, 4= Don't know
20. What has been the trend of rainfall for the past 30 years in the area?	1=Increase, 2=decrease, 3= Not change, 4= Don't know
21. What changes you observed in rainfall over the past 20 Years	1=Increase, 2=decrease, 3=Not change, 4= Don't know
22. Is rainfall coverage during the rainy season increased?	1=Yes, 0=No
23. Do you think that the occurrence of drought over 20 years increased	1=Yes, 0=No
24. Have you observed variation in winter rainfall	1=Yes, 0=No
25. Is rains sometimes comes late and cease early in your kebele?	1=Yes, 0=No
26. Do you think that crop disease/ pest outbreaks increased over 20 years?	1=Yes, 0=No
27. Is the frequency of observed drought in numbers increased over the last 20 years?	1=Yes, 0=No
28. Did you get information about early warning about natural disasters timely	1=Yes, 0=No
29. The occurrence of Livestock disease outbreaks increased over 20 years.	1=Yes, 0=No

30. Have you received any training about climate? 1= Yes, 0= No
31. Are you using a weather forecast for your farming decisions? 1= Yes, 0= No
32. Have you observed any drought occurrences over the last five years? 1= Yes, 0= NO
33. If YES, how many times has this area been affected by a drought between 2009–2013E. C? ---
34. Have you observed any flooding occurrences over the last five years? 1= Yes, 0= NO
35. If yes, how many times has this area been affected by flooding between 2010–2015E. C? ------
36. In the last 5 years, what physical assets have you lost or had severely damaged due to flooding/high wind events? ....
37. Did you receive a warning about the most severe flood /drought event in the past 5 years before it happened? 1= Yes, 0= NO,

D) Agricultural system and natural resource

38. Do you perceive rapid natural vegetation conversions in your area?1= yes, 2= No
39. Do you believe your farm plot is fertile for production? 1. Yes 2. No
40. Do you perceive rapid land degradation in your area? 1= yes 2= No
41. Are you an active member of any group/organization/farmers' cooperation/farmers' club?

E) Livelihood Strategies/Sources of Household Income

42. Do you have dwelling house constructed from wood and corrugated iron? 1=Yes 2=No
43. Is the household head house will be unable to withstand storm surges (high wind) and floods? 1=Yes, 0=No
44. How much money did you earn from last year's (i.e. 2015 EC) crop sale _____________ (ETB)
45. Do you have your own livestock? 1. Yes 2. No
46. What type of livestock do you own, and what are their numbers?

	Livestock you have								
No	Cattle	Goat	Sheep	Donkey	Horse	Mule	Poultry	Others (specify)	Don't have
Number									

47. How much money did you earn from last year's (i.e. 2015 EC) livestock and livestock product sale _______________ (ETB)
48. Family members working as active labor on farm (numbers)______________
49. Have you or a member of the household engaged in off-farm activities? 1. Yes 2. No
50. What is your average off-farm income/month__________?
51. Annual average income from honey and related products in Ethiopian birr____________.
52. Please indicate the types of livelihood activities carried out? (*Multiple answers are possible*)

1. Agriculture	1=Yes, 0=No
2. Timber, fuel wood and charcoal	1=Yes, 0=No
3. Tourism employment	1=Yes, 0=No
4. Daily labor	1=Yes, 0=No
5. Handcraft	1=Yes, 0=No
6. Providing transport services (car, Bajaj, etc.)	1=Yes, 0=No

F) Social Networks

53. During the past month, did relatives or friends help you and your family? 1=Yes, 0=NO
54. Did you and your family help relatives or friends in the past month? 1=Yes, 2= No
55. If yes, list the types of help given by you to another household in the past ______
56. Did you borrow any money from relatives or friends in the past three months?
    1= Yes, 0= NO
57. Did you lend any money to relatives or friends in the past three months? 1= Yes, 0= NO
58. In the past 12 months, have you or another member of your household gone to your local government office/ official for help? 1=Yes, 0=No
59. Are you a member of social institutions in your village? 1=Yes, 0= No
60. If YES, the members of which local institution/s? 1= *Iddir*, 2=*Equb*, 3= Religious groups, 4= None
61. Are you a member of farmers' group/cooperative? 1=Yes, 0= No
62. Do you have access to credit from savings and credit institutions? 1= Yes, 0= No
63. Did you borrow any money from credit institutions in the last year? 1= Yes, 0= No
64. If yes, for what purpose? ____
65. If no, why? ____

G) Water Sources

66. What is /are the main water sources for drinking and cooking for members of your households? _____
67. How long does it take to get to your main water source? ______(walking minutes/hours)
68. Is this water available every day and every month? 1=Yes, 0=No
69. During the past year, have you heard about any conflicts over water in your community?
    1=Yes, 0=No

H) Knowledge/ Skill

70.	Have you received any training over the 1 years?	1=Yes, 0=No
71.	if your answer is yes for QN 70, what type of training you have attended over the last 1 year?	
1	Training to cope with climate change	1=Yes, 0=No
2	Training on health extension service	1=Yes, 0=No
3	Training on farm management	1=Yes, 0=No
4	HHs who received training to cope with climate change	1=Yes, 0=No
72.	Do you have farmer-to -farmer extension platform on the use decision and success of agricultural technologies?	1=Yes, 0=No
73.	Are you a member of any input or seed or market cooperatives?	1=Yes, 0=No
74.	Do you have land use right or ownership certificate?	1=Yes, 0=No

I) Technology

75. Have you used improved seed? 1=Yes, 0=No
76. Have you used artificial fertilizers? 1=Yes, 0=No
77. Have you used insecticide, herbicide and pesticide? 1=Yes, 0=No
78. Have you applied rain-water harvesting? 1=Yes, 0=No
79. Do you have practice of irrigation? 1=Yes, 0=No
80. Does the family own a radio or/and a telephone at home? 1=Yes, 0=No

J) Infrastructure and Information Access

81. Are there roads that connect the *Kebele* you with nearby towns or cities? 1=Yes, 0=No
82. What is the distance to the nearest main road from your home? (walking minute) ____
83. What is the distance from your home to the nearest school? (walking minutes)
84. Do you have market access nearby? 1=Yes, 0=No
85. Time taken to reach the nearest market where you sell your harvest or livestock products _
86. Do your *Kebele* development agent/s offer agricultural extension services? 1=Yes, 0=No
87. What is the distance from your home to extension services in minutes? _______
88. What is the walking distance to veterinary services in minutes? _____________
89. Do you have health center nearby? 1=Yes, 0=No
90. What is the walking distance from home to health center on foot in minutes? _______
91. What is the walking distance from home to credit and saving institutions in in minutes? _____
92. Do you have access to agricultural inputs? 1=Yes, 0=No
93. Do you use agricultural inputs? 1=Yes, 0=No
94. Do you use an energy efficient Stove? 1=Yes, 0=No

Lastly, I thank you for your cooperation!
